# Influence of Acetylcholinesterase Inhibitors Used in Alzheimer’s Disease Treatment on the Activity of Antioxidant Enzymes and the Concentration of Glutathione in *THP-1* Macrophages under Fluoride-Induced Oxidative Stress

**DOI:** 10.3390/ijerph16010010

**Published:** 2018-12-20

**Authors:** Marta Goschorska, Izabela Gutowska, Irena Baranowska-Bosiacka, Katarzyna Piotrowska, Emilia Metryka, Krzysztof Safranow, Dariusz Chlubek

**Affiliations:** 1 Department of Biochemistry and Medical Chemistry, Pomeranian Medical University in Szczecin, Powst. Wlkp. 72, Szczecin 70-111, Poland; irena.bosiacka@pum.edu.pl (I.B.-B.); emilia_metryka@o2.pl (E.M.); chrissaf@mp.pl (K.S.); dchlubek@pum.edu.pl (D.C.); 2 Department of Biochemistry and Human Nutrition, Pomeranian Medical University in Szczecin, Broniewskiego 24, Szczecin 71-460, Poland; izagut@poczta.onet.pl; 3 Department of Physiology, Pomeranian Medical University in Szczecin, Powst. Wlkp. 72, Szczecin 70-111, Poland; katarzyna.piotrowska@pum.edu.pl

**Keywords:** donepezil, rivastigmine, fluoride, macrophages, antioxidant enzymes, reactive oxygen species

## Abstract

It has been reported that donepezil and rivastigmine, the acetylcholinesterase (AchE) inhibitors commonly used in the treatment of Alzheimer’s disease (AD), do not only inhibit AChE but also have antioxidant properties. As oxidative stress is involved in AD pathogenesis, in our study we attempted to examine the influence of donepezil and rivastigmine on the activity of antioxidant enzymes and glutathione concentration in macrophages—an important source of reactive oxygen species and crucial for oxidative stress progression. The macrophages were exposed to sodium fluoride induced oxidative stress. The antioxidant enzymes activity and concentration of glutathione were measured spectrophotometrically. The generation of reactive oxygen species was visualized by confocal microscopy. The results of our study showed that donepezil and rivastigmine had a stimulating effect on catalase activity. However, when exposed to fluoride-induced oxidative stress, the drugs reduced the activity of some antioxidant enzymes (Cat, SOD, GR). These observations suggest that the fluoride-induced oxidative stress may suppress the antioxidant action of AChE inhibitors. Our results may have significance in the clinical practice of treatment of AD and other dementia diseases.

## 1. Introduction

The brain is particularly sensitive to reactive oxygen species (ROS) as a result of its very intense metabolism and low regenerative capacity [1] in comparison with other tissues. Despite the low weight it processes 20% of basal O_2_ consumption [2], using oxygen for transformations in mitochondria in order to obtain ATP, necessary to maintain a low gradients of ions or in glucose metabolism to obtain energy for neurons [2]. 

It has been shown that oxidative stress is associated with neurodegenerative diseases such as Alzheimer's disease (AD), Parkinson's disease (PD) and amyotrophic lateral sclerosis (ALS). In addition, post mortem examination of patients with these diseases have shown that the regions of the brain affected by neurodegeneration displayed increased ROS indices [1,3,4,5].

Participating in the body's response to various pathogenic factors, macrophages play a key role in inflammation, and constitute the main source of ROS in the human body. Although it has been previously thought that ROS are mainly produced by resident macrophages in the brain, i.e. microglia, recent reports also indicate the important role of peripheral cells, especially macrophages, which points to their significance for the modulation and progression of inflammation [6]. A particularly important source of ROS is activated macrophages, and their increased production may adversely affect the pro-oxidant–antioxidant balance [7]. 

The protection of cells against the effects of excessive oxidation depends on the action of antioxidant enzymes such as superoxide dismutase (SOD, EC 1.15.1.1.1), superoxide peroxidase (Gpx, EC 1.11.1.9), superoxide reductase (GR, EC 1.6.4.2) and catalase (CAT, EC 1.11.1.6) [8,9]. Their neuroprotective action has a proven role in supporting the treatment of neurodegenerative diseases (including Alzheimer's disease) and slowing down the disease process [10]. 

SOD catalyzes the dismutation of superoxide to hydrogen peroxide [11]. The resulting hydrogen peroxide is then decomposed and catalyzed by CAT. H_2_O_2_ may also be reduced in a reaction catalyzed by GPx, a selenoprotein that has the ability to reduce not only hydrogen peroxide, but also other inorganic and organic peroxides (including lipid peroxides) [12]. The availability of glutathione in the reduced form (GSH) is essential for hydrogen peroxide decomposition catalyzed by GPx, associated with with glutathione oxidation (GSSG) occurs and H_2_O_2_ reduction [13,14]. In order for the GPx-catalyzed reaction to run smoothly, it is necessary to reconstruct GSH. This glutathione reduction reaction is catalyzed by GR, with NADPH serving as the electron donor [13]. GSH not only acts as a cofactor of the aforementioned enzymes, but also has the ability to interact directly with ROS [8]. Therefore, the proper functioning of the antioxidant system requires the participation of all the mentioned antioxidant enzymes and glutathione, and any disturbances in their activity or amount may cause oxidative stress [8,13,14]. 

As mentioned earlier, AD is an example of a chronic neurodegenerative disease with progressive course in which imbalance between ROS formation and elimination is one of the pivotal factors [1,15]. Currently, the symptomatic treatment of AD and different kinds of dementias commonly involves acetylcholinesterase inhibitors usage [16]. In the USA, cholinesterase inhibitors are the only approved pharmacological treatment for Alzheimer's disease that have appropriate efficacy [15]. They are medicaments widely used in the pharmacotherapy AD symptoms, at different stages of advancement. Their action consists in inhibiting at least one of the enzymes catalyzing the hydrolysis of acetylcholine (ACh) or butyrylcholine (BuChE). The action of the inhibitors results in an elevation in the quantity accessible ACh and in enhancement of synaptic transmission [17]. Rivastigmine, donepezil or galantamine belong to medicaments inhibiting AChE. They are used to treat various dementias (including AD and vascular dementia) [18,19]. 

Donepezil is one of the most commonly used medicines to treat dementia. It inhibits AChE with high selectivity and in a non-competitive manner [16,20], is able to cross the blood–brain barrier (BBB) [21]. Its long-term usage is possible thanks to good tolerance and slow elimination from the human body [16]. Donepezil's mechanism of action is reported to be mainly related to AChE inhibition, but also other properties have been recently mentioned, such as stimulation of the cholinergic transmission, which protects against inflammation (although not influencing BuChE) [16]. Donepezil-dependent defense of microglia against inflammatory processes was also demonstrated in models without ACh. That suggests more complicated way of action of this drug [22]. 

Rivastigmine, a long-acting drug, inhibits BuChE or AChE in a quasi-irreversible and not competitive way [23]. Rivastigmine is able to penetrate through the brain protecting barrier (BBB) [16,24]. It induces the inhibition of AChE and BuChE by carbamylation of serine in the catalytic triad [25]. However, the mechanism of the long-term action of rivastigmine is not entirely clear, for example the causes of a significant upregulation of AChE expression [26]. There are also reports on the anti-inflammatory properties of rivastigmine such as the reduced production of cytokines and inhibition of encephalitogenic T lymphocyte reactivity [27], with the exact mechanism undefined and requiring further research, similar to donepezil [16].

Fluoride (F) is an element with proven prooxidative properties and an ability to cross the BBB. It can induce neuroinflammation and neurodegeneration which may be clinically manifested as memory, concentration or cognitive disorders [28,29]. The most important toxic effects of F in the brain include increase in prooxidative processes rate with subsequent damage to neurons, impairment of signal transmission within synapses, and induction of inflammation [30]. Mentioned ravages concerning fluoride action may possibly be in relation to nervous tissue degenerative changes reported in AD [31].

Fluoride exerts the inhibitory influence on various enzymes’ expressions and activities. Cholinesterases belong to the enzymes with their activities being inhibited after fluoride exposure [32]. The arresting effect of fluoride compounds has been reported for at least for tens of years. In 1985, Baselt et al. reported decreased cholinesterase activity in postmortem, fluoride preserved blood samples in comparison to the postmortem blood samples with no fluoride preservation [33]. Currently many studies are focused on the methane sulfonyl fluoride (MSF) examination, because of its acetylcholinesterase inhibitory properties in the irreversible manner [34]. MSF attaches the enzyme (AChE) catalytic site solidly, entirely irreversibly [34] and without any possibility of spontaneous hydrolysis of the covalent MSF-ACh bond [35] MSF exerts the selective inhibitory properties on brain AChE [36]. 

Macrophages obtained after *THP-1* monocytes transformation can be recognized as a simplified investigational simulation to study the effect of rivastigmine as well as donepezil on antioxidant enzyme activity and GSH concentration in brain microglial cells [37,38]. This is because microglial dysfunction increases the severity of symptoms and accelerates the progress of age–related neurodegenerative diseases, such as Alzheimer's disease [39]. *THP-1* macrophages may constitute a good experimental model to study the mechanisms of macrophages in atherosclerosis, and vascular dementia [40], while the model of the proinflammatory and pro-oxidant effects of F on macrophages has already been used in our earlier works and in the works of other authors [28,41].

The aim of the recent paper is to determine the effect of donepezil and rivastigmine on the activity of antioxidative enzymes (SOD, CAT, GPx, GR) and the concentration of GSH in macrophages generated from the *THP-1* cell line monocytes using the model of pro-oxidative effect of fluoride.

## 2. Materials and Methods

### 2.1. Reagents

Sigma-Aldrich (Poland) was a supplier of: RPMI-1640 medium, amino acid (glutamine), antibiotics such as streptomycin or penicillin, phorbol myristate acetate (PMA), sodium fluoride (NaF), dimethyl sulfoxide (DMSO), rivastigmine and donepezil. Assay kits used for determination of examined enzymes activities and glutathione quantity were obtained from Cayman Chemical (USA). Bakerbond extraction columns were obtained from JT Baker (USA). PBS (phosphate buffer saline) was obtained from PAB Laboratories (Vienna, Austria). Fetal bovine serum (FBS) was purchased from Gibco Invitrogen (Holland). Small laboratory supplies were bought from Becton-Dickinson (USA), Sarstedt (Germany) or Applied Biosystems (USA). American Type Culture Collection (USA) provided monocytes of *THP-1* line. 

### 2.2. Cultivation and Treatment of Cells

*THP-1* monocytes were grown in the Roswell Park Memorial Institute medium 1640 (Sigma-Aldrich, Poland) enriched with FBS (10%), free of fatty acids (FBS; GIBCO, Holland), and enriched with antibiotic (100 U/ml of penicillin and 100 mg/ml of streptomycin) (Sigma-Aldrich, Poland). The cell cultivation was conducted at 37 °C in 5% CO_2_. Viability of the monocytic cells implemented in experiments was analyzed with the usage of Trypan blue, Bright-Line Hemacytometer (purchased from Hausse Scientific, USA) and a microscope (Olympus M021, USA). Monocytes exhibiting viability higher than 95% were chosen to analyze [42,43]. Cells were subsequently placed in the six-well cultivation plates and activated into macrophages by adding a 100 nM solution of PMA to the medium [43]. Monocyte culture with PMA was carried out for 24 h. The adherent macrophages obtained were washed thrice with PBS (PAB Laboratories, Austria), then cultured for 48 h with donepezil or rivastigmine solutions at specific concentrations and combinations as in Table 1 and Table 2. The same experiment was performed in macrophages (*THP-1*) exposed to sodium fluoride, which exerts pro-oxidant effects (Table 3). The concentration of NaF was 3 µM per single well.

Table 1 shows the concentrations of particular medicaments used in this study. Selection of the AChIs concentrations was conducted in relation to the concentrations values reported within the blood serum of people taking the minimal and the maximal allowed doses [44,45,46,47,48,49].

The applied model of the effect of NaF on macrophages has been previously described, and the results showed a pro-oxidant and promoting inflammation action [50,51,52,53]. 

### 2.3. Enzyme Activity

The following reagent kits were used to determine antioxidant enzyme activity: Superoxide Dismutase Assay Kit (Cayman Chemical, Ann Arbor, MI, USA), Catalase Assay Kit (Cayman Chemical, Ann Arbor, MI, USA), Glutathione Peroxidase Assay Kit (Cayman Chemical, Ann Arbor, MI, USA), Glutathione Reductase Assay Kit (Cayman Chemical, Ann Arbor, MI, USA). The determinations were made spectrophotometrically in accordance with the protocols provided by the manufacturers.

### 2.4. Glutathione (GSH) Concentration

A Glutathione Assay Kit (Cayman Chemical, Ann Arbor, MI, USA) was performed to determine the concentration of the reduced form of glutathione. The determination was made by spectrophotometric method according to the procedure provided by the supplier.

### 2.5. Fluorescent Studies

#### Visualization and Quantitative Estimation of ROS Formation Within the Cells

The imaging of ROS synthesis within the cells was performed with the use of luminescent indicator 2′,7′-dichlorofluorescein diacetate (DCFH-DA) (Sigma-Aldrich, Poland) [54,55,56]. Macrophages were stuffed with DCFH-DA (5 μM). After the exposition had been terminated, macrophages were washed thrice with cultivation medium at ambient temperature. Confocal microscope was used to analyze the obtained preparations. H_2_O_2_-dependent oxidation of DCFH-DA is accompanied by fluorescence (excitation at 495 nm, emission at 525 nm). 

To evaluate the amount of ROS produced in the cytosol, the examined cells underwent pre-treatment with luminescent marker in the conditions like described in the previous sentence. DCF-dependent signal and its strength was detected by microplate reader. In the next step the results were converted in relation to protein amount. MicroBCA assay was performed in order to measure the sample protein value [57,58].

### 2.6. Protein Assay

All the above-mentioned results were calculated from the protein content in the samples. Protein concentration was measured using a MicroBCA Protein Assay Kit (Thermo Scientific, Pierce Biotechnology, USA) and plate reader (UVM340, ASYS) [59]. 

### 2.7. Statistic Evaluation

To analyze the results software from StatSoft (Poland)—Statistica 10 was used. The dependent variables analysis was conducted with use of the Shapiro–Wilk W-test. In calculations nonparametric tests were performed. The arithmetical mean ± standard deviation (SD) was performed to express the results. A *p*-value ≤ 0.05 was recognized as significant.

## 3. Results

### 3.1. Effect of Donepezil and Rivastigmine on Intracellular ROS Generation in Macrophages

#### Rivastigmine and Donepezil Inhibited ROS Generation in Macrophages

**Figure 1:**
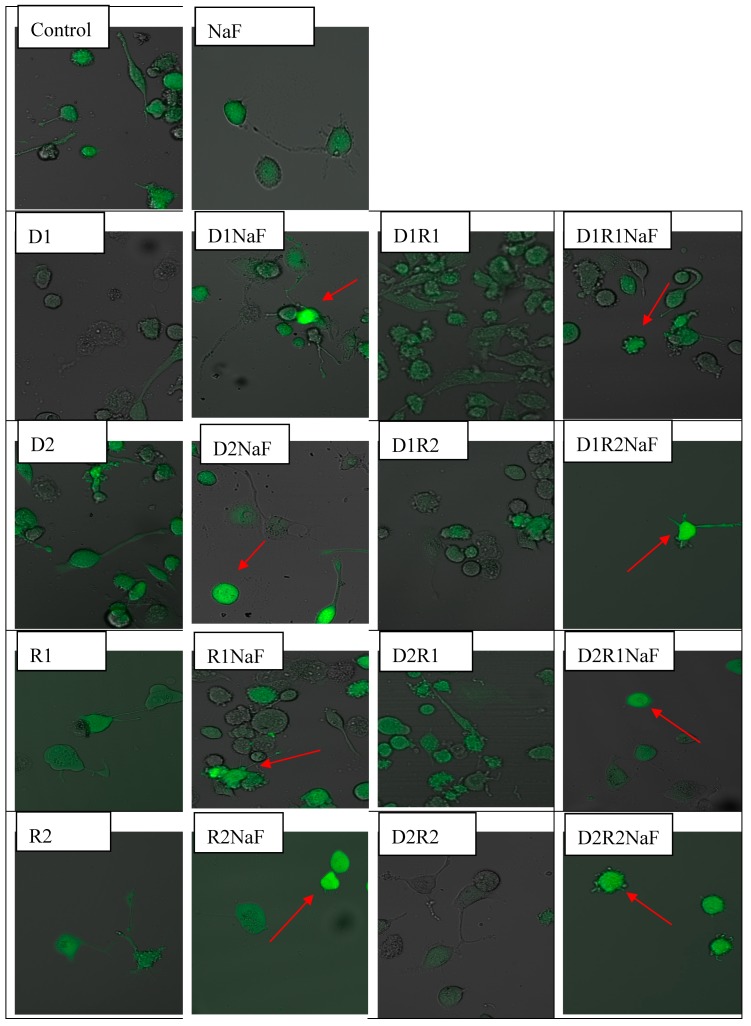
Formation of intracellular reactive oxygen species (ROS) imaged by confocal microscopy in *THP-1* macrophages exposed to donepezil and/or rivastigmine; and in cells simultaneously exposed to fluoride.

Microscopic studies showed the same green fluorescence level coming from DCF (thereby ROS generation in the cytoplasm of macrophages) from donepezil and/or rivastigmine-treated cells vs control (Figure 1). Calculations concerning the fluorescence exertion demonstrated the lack of differences in ROS amount within drug-treated macrophages vs control cells (Table 1).

### 3.2. The Effect of Donepezil and Rivastigmine on Intracellular ROS Generation in Fluoride-Exposed Macrophages

#### Prooxidative Fluoride Condition Increased ROS Quantity in Macrophages

Analyzes of microscopic images of macrophages cultured with rivastigmine and/or donepezil in fluoride-exposed macrophages showed intensified ROS generation (in cytosol) in comparison to control cells (DCF was the source of green fluorescence) (Figure 1). Increased ROS formation in macrophages cytoplasm vs control (in all studied conditions) was confirmed after the intensity of fluorescence evaluation (Table 4).

Cells were treated with rivastigmine and donepezil or with both medicaments. Final concentration of donepezil was 20 ng/mL (D1) or 100ng/mL (D2) per single well. Concentration of rivastigmine in an incubation well was concentration of 5 ng/mL (R1) ml or 25 ng/mL (R2). Concentration of sodium fluoride was 3 μM in a single well. Culture of macrophages together with NaF and DMSO served as the control. Exposition of macrophages to medicaments (D, R or DR) lasted 48 h. The intracellular generation of ROS was visualized by fluorescent indicator 2′,7′-dichlorofluorescein diacetate (DCFH-DA). Cells were filled with DCFH-DA used at the concentration of 5 μM. Following 15 min lasting exposure, macrophages were rinsed with use of medium at ambient temperature. Confocal microscope was used to analyze the obtained microscopic preparations. DCFH-DA dependent fluorescence (excitation at 495 nm, emission at 525 nm) appears as the result of its intracellular oxidation by H_2_O_2_, the red arrow indicates increased ROS level vs control group. (color should be used in print)

Cells were incubated with 5 μM of DCFH-DA. A microplate reader was used to estimate the intracellularly generated ROS. Fluorescence exertion was evaluated in relation to protein quantity, performed by Bradford method. 

In summary, under fluoride-induced oxidative stress we observed a statistically significant higher concentration of ROS in the cytoplasm of macrophages incubated with donepezil and rivastigmine at all tested concentrations and combinations of the drugs in comparison to control.

### 3.3. Donepezil and Rivastigmine Exerted Influence on Superoxide Dismutase Activity (SOD) in Macrophages

#### 3.3.1. Rivastigmine and Donepezil did not Affect SOD Activity in Macrophages

**Figure 2:**
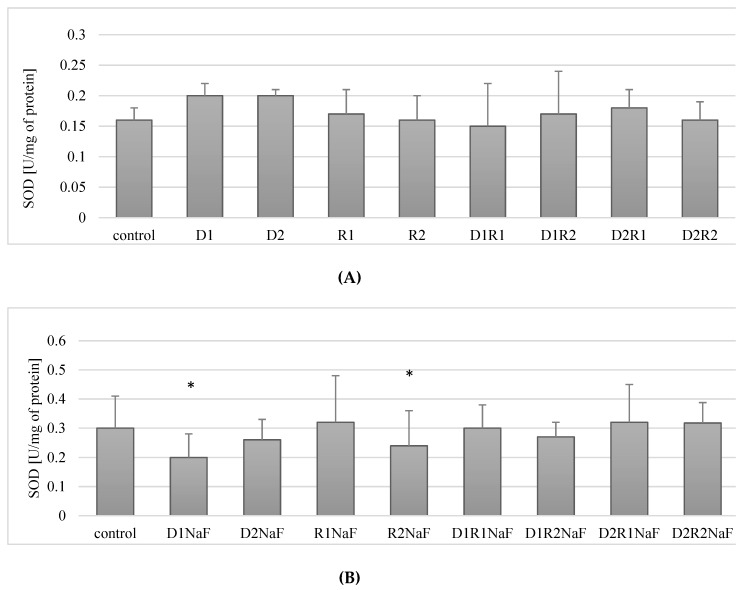
Influence of donepezil and rivastigmine on superoxide dismutase (SOD) activity in *THP-1* macrophages (**A**) and in fluoride-exposed *THP-1* macrophages (**B**).

The addition of donepezil at either 20 ng/mL (D1) or 100 ng/mL (D2) did not affect SOD activity compared to control. Changes in SOD activity were also not observed in cells cultured with rivastigmine at either 5 ng/mL (R1) or 25 ng/mL (R2). Combined implementation of the drugs also did not significantly affect the activity of SOD within the macrophages cultured with any of the concentrations used (D1R1, D1R2, D2R1, D2R2) (Figure 2A). 

In summary, the drugs used in the study (both separately and in combination) had no effect on the activity of SOD in THP-1 macrophages.

#### 3.3.2. Rivastigmine and Donepezil Used Separately Inhibited SOD Activity in Fluoride-Exposed Macrophages

It was shown that incubation of fluoride-exposed cells with donepezil at 20 ng/mL (D1) resulted in a statistically significant reduction in SOD activity compared to control of about 30% (*p* = 0.05) (Figure 2B). Cultivation of cells with donepezil (100 ng/mL; D2) did not influence SOD activity when compared to control (*p* = 0.07) (Figure 2B).

The use of rivastigmine at 5 ng/mL (R1) did not cause statistically significant changes in SOD activity compared to control (*p* = 0.34). However, a significant decrease (by approx. 17%) in enzyme activity was observed in macrophages incubated with rivastigmine at the higher concentration of 25 ng/mL (R2) compared to the control (*p* = 0.02) (Figure 2B).

When fluoride-exposed macrophages were treated with donepezil and rivastigmine together at concentrations: 20 ng/mL and 5 ng/mL (D1R1), 20 ng/mL and 25 ng/mL (D1R2), 100 ng/mL and 5 ng/mL (D2R1) as well as 100 ng/mL and 25 ng/mL (D2R2), it did not cause significant changes in SOD activity compared to control (*p* = 0.7, *p* = 0.12, *p* = 0.35 and *p* = 0.25, respectively) (Figure 2B).

In summary, fluoride-exposed macrophages incubated with donepezil at 20 ng/mL or rivastigmine at 25 ng/mL showed a statistically significantly decreased SOD activity compared to control. In the other experimental condition, no statistically significant changes in SOD activity were observed.

Donepezil was used at 20 ng/mL (D1) or 100 ng/mL (D2). Concentrations of rivastigmine were respectively 5 ng/mL (R1) or 25 ng/mL (R2). DMSO-treated cells served as a control. In a model of fluoride-induced oxidative stress NaF was implemented at a concentration of 3 μM and macrophages incubated with NaF and DMSO served as a control. Cells were cultured with acetylcholinesterase inhibitors for 48 h. After incubation cells were harvested by scraping and SOD activity was estimated spectrophotometrically using Superoxide Dismutase Assay Kit (Cayman Chemical, USA). Data represent means ± SD for 6 independent experiments. * *p* < 0.05, statistically significant differences versus control using Wilcoxon test. 

### 3.4. Donepezil and Rivastigmine Modulation of Catalase Activity (CAT) in Macrophages

#### 3.4.1. Rivastigmine as well as Donepezil Used Separately Increased the Activity of CAT in Macrophages

**Figure 3:**
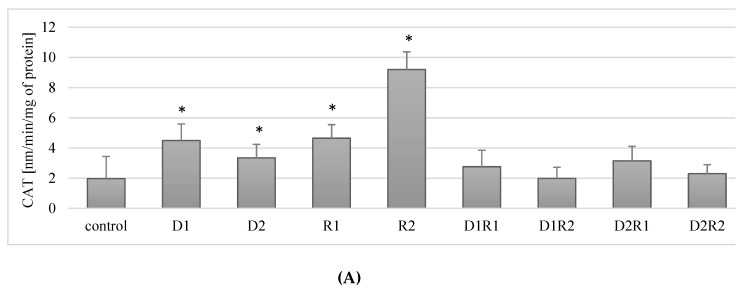

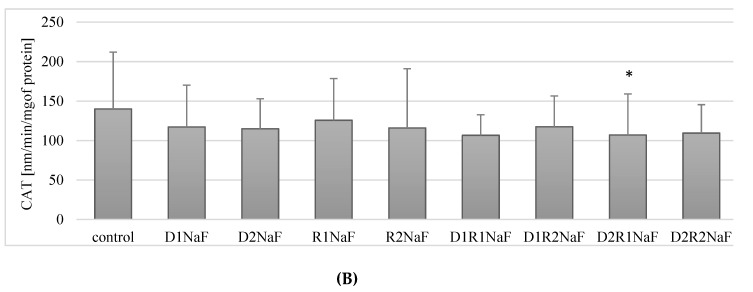
Influence of donepezil and rivastigmine on catalase (CAT) activity in *THP-1* macrophages (**A**) and in fluoride-exposed *THP-1* macrophages (**B**).

In cells incubated with donepezil, a statistically significant increase in CAT activity was observed compared to the control for both concentrations used (respectively: for 20 ng/mL (D1) by about 188% (*p* = 0.04), for 100 ng/mL (D2) by ca. 70% (*p* = 0.04)). Similar relationships were observed for rivastigmine: at 5 ng/mL (R1) an increase of about 136% (*p* = 0.04) and at 25 ng/mL (R2) by 367% (*p* = 0.04) compared to the control (Figure 3A).

Combined use of the drugs at the lower concentrations (D1R1) resulted in a 40% increase in CAT activity in *THP-1* macrophages. However, the difference was not statistically significant (*p* = 0.2). When using the other concentrations (D1R2, D2R1, D2R2), no statistically significant changes in CAT activity were observed relative to the control (Figure 3A).

In conclusion, the use of rivastigmine and donepezil separately at both lower and higher concentrations resulted in an increase in CAT activity compared to controls. The combined use of the drugs did not have a statistically significant effect on CAT activity in the macrophages.

#### 3.4.2. Rivastigmine and Donepezil Inhibited CAT Activity in Fluoride-Exposed Macrophages

Fluoride-exposed macrophages showed no statistically significant differences in CAT activity following the incubation of macrophages with donepezil at 20 ng/mL (D1) (*p* = 0.07) and 100 ng/mL (D2) (*p* = 0.12) (Figure 3B) and using rivastigmine at 5 ng/mL (R1; *p* = 0.75) and 25 ng/mL (R2; *p* = 0.12) compared to the control. 

A statistically significant (*p* = 0.04) decrease in CAT activity of approx. 24% in relation to the control was observed in macrophages exposed to donepezil and rivastigmine, used together, at concentrations of 100 ng/mL and 5 ng/mL, (D2R1) (Figure 3B).

The use of donepezil and rivastigmine in the other examined combinations (D1R1, D1R2, D2R2) did not have a statistically significant effect (each *p* = 0.12) on CAT activity in fluoride-exposed macrophages in relation to the control. 

In summary, in fluoride-exposed macrophages there was a statistically significant lower CAT activity compared to controls, when incubated with donepezil at 100 ng/mL together with rivastigmine at 5 ng/mL. In other cell cultures, i.e. those incubated with: D1, D2, D1R1, D1R2, D2R2, there was no statistically significant effect of the drugs on CAT activity in fluoride-exposed macrophages (Figure 3A,B).

Donepezil was used at 20 ng/mL (D1) or 100 ng/mL (D2). Second medicament-rivastigmine was added at concentrations: 5 ng/mL (R1) mL or 25 ng/mL (R2). Culture of macrophages together with DMSO was treated as a control. In a model of fluoride-induced oxidative stress, sodium fluoride was added (3 μM). As a control cells cultivated with addition of NaF and DMSO were set. Cells were cultured with acetylcholinesterase inhibitors for 48 h. After incubation cells were harvested by scraping and CAT activity was estimated spectrophotometrically using a Catalase Assay Kit (Cayman Chemical, USA). Data show means ± SD for six separate experiments. * *p* < 0.05, differences being statistically significant in comparison to control using Wilcoxon test.

### 3.5. Effect of Donepezil and Rivastigmine on Glutathione Peroxidase (GPx) Activity in Macrophages

#### 3.5.1. Rivastigmine and Donepezil Did Not Affect GPx Activity in Macrophages

**Figure 4:**
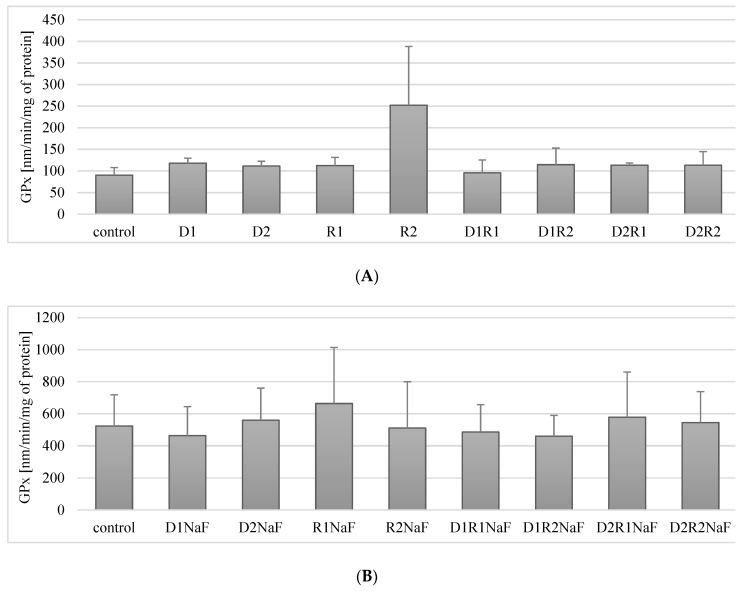
Influence of acetylcholinesterase inhibitors donepezil and rivastigmine on glutathione peroxidase (GPx) activity in *THP-1* macrophages (**A**) and in fluoride-exposed *THP-1* macrophages (**B**).

Cultivation of the cells with donepezil at 20 ng/mL (D1) or 100 ng/mL (D2), did not result in statistically significant differences in GPx activity in relation to control (*p* = 0.07 and *p* = 0.2, respectively). Similar non-significant relationships were also observed with rivastigmine at 5 ng/mL (R1; *p* = 0.07) or 25 ng/mL (R2; *p* = 0.07) (Figure 4A). 

Macrophages exposure to the combined use of therapeutics in the studied systems (D1R1, D1R2, D2R1, D2R2) also had a statistically insignificant effect on GPx activity in macrophages relative to the control (Figure 4A).

In summary, the use of acetylcholinesterase inhibitors, donepezil and rivastigmine, did not have a statistically significant effect on GPx activity in macrophages.

#### 3.5.2. Rivastigmine and Donepezil Did Not Affect GPx Activity in Fluoride-Exposed Macrophages

In prooxidative condition of sodium fluoride (NaF) on macrophages, it was shown that cells incubation both in the presence of donepezil at 20 ng/mL (D1) or 100 ng/mL (D2) did not affect GPx activity relative to the control (*p* = 0.35) (*p* = 0.46) (Figure 4B). 

Similarly, in macrophages incubated with rivastigmine at both concentrations: R1 and R2 no statistically significant changes in GPx activity were observed compared to control (*p* = 0.17 and *p* = 0.46). 

The combined use of both drugs: donepezil and rivastigmine in the studied systems (D1R1, D1R2, D2R1, D2R2) did not significantly influence the change in GPx activity as compared to the control (respectively: *p* = 0.25, *p* = 0.12, *p* = 0.6, *p* = 0.25) (Figure 4B).

In conclusion, in fluoride-exposed macrophages, the separate and combined use of donepezil and rivastigmine did not affect GPx activity at any of their concentrations studied.

Donepezil was used at 20 ng/mL (D1) and 100 ng/mL (D2). Concentrations of the rivastigmine used were respectively: 5 ng/mL (R1) and 25 ng/mL (R2). Control group comprised of DMSO-exposed macrophages. In a conditions of fluoride prooxidative action NaF was used at 3μM and macrophages cultivated with both incubated with NaF and DMSO were used as a control. Cells were cultured with acetylcholinesterase inhibitors for 48h. After incubation cells were harvested by scraping and GPx activity was estimated spectrophotometrically using Glutathione Peroxidase Assay Kit (Cayman Chemical, USA). Data describes means ± SD for 6 separately conducted experiments. * *p* < 0.05 value represents differences that were significant versus control, estimated by Wilcoxon test.

### 3.6. Influence of Donepezil and Rivastigmine on Glutathione Reductase (GR) Activity in Macrophages

#### 3.6.1. Rivastigmine and Donepezil Did Not Affect GR Activity in Macrophages

**Figure 5:**
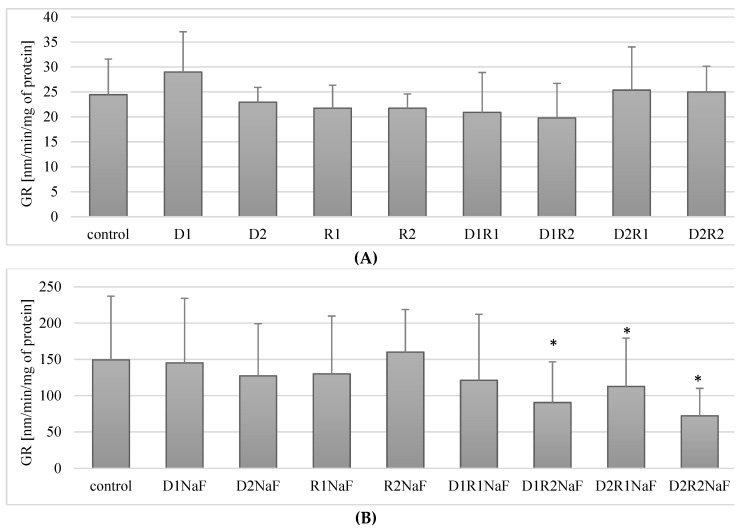
Influence of acetylcholinesterase inhibitors on glutathione reductase (GR) activity in *THP-1* macrophages (**A**) and fluoride-exposed *THP-1* macrophages (**B**).

Incubation of the studied macrophages with donepezil at 20 ng/mL (D1) or100 ng/mL (D2) did not have a statistically significant effect on GR activity compared to the control (*p* = 0.68; *p* = 0.9 appropriately). Similarly, the use of rivastigmine at 5 ng/mL (R1) and 25 ng/mL (R2) did not cause statistically significant differences in GR activity compared to controls (*p* = 0.2; *p* = 0.34 appropriately) (Figure 5A). 

No statistically significant differences in GR activity compared to controls were observed in macrophages treated with combinations of donepezil and rivastigmine in the combinations tested.

In summary, the use of acetylcholinesterase inhibitors donepezil and rivastigmine both separately and in combination did not have a statistically significant effect on GR activity in macrophages.

#### 3.6.2. Rivastigmine and Donepezil Decreased GR Activity in Macrophages in Fluoride-Exposed Macrophages

In fluoride-exposed macrophages, no statistically significant differences in GR activity were observed compared to controls with donepezil applied at either 20 ng/mL (D1; *p* = 0.6) and 100 ng/mL (D2; *p* = 0.46). Similar relationships were noted for both tested rivastigmine concentrations (R1 and R2) (Figure 5B).

The exposure of macrophages to fluoride and to the combined drugs donepezil and rivastigmine at 20 ng/mL and 5 ng/mL respectively (D1R1) also did not have a statistically significant effect on GR activity compared to controls (*p* = 0.6) (Figure 5B). However, the use of all other combinations of donepezil and rivastigmine resulted in a significant reduction in enzyme activity with respect to control. The D1R2 concentration system caused a reduction of GR activity by approx. 40% compared to the control (*p* = 0.02), D2R1 decreased the enzyme activity by ca. 62% (*p* = 0.04), and the D2R2 system by ca. 52% compared to controls (*p* = 0.02) (Figure 5B).

In fluoride-exposed macrophages, statistically significantly lower GR activity was observed compared to the control in the cells incubated together with the studied drugs in the following systems: donepezil 20 ng/mL and rivastigmine 5 ng/mL (D1R2), donepezil 100 ng/mL and rivastigmine 5 ng/mL (D2R1) and in macrophages cultured with the combination of these two drugs at the maximum concentrations used (D2R2).

Donepezil was used at 20 ng/mL (D1) and 100 ng/mL (D2). Rivastigmine was used at 5 ng/mL (R1) and 25 ng/mL (R2). Macrophages incubated with DMSO were used as the control. In a model of fluoride-induced oxidative stress, NaF was used at 3 μM and macrophages incubated with NaF and DMSO were used as a control. Cells were cultured with acetylcholinesterase inhibitors for 48 h. After incubation cells were harvested by scraping and GR activity was estimated spectrophotometrically using a Glutathione Reductase Assay Kit (Cayman Chemical, USA). Data represent means ± SD for six independent experiments. * *p* < 0.05, statistically significant differences versus control using Wilcoxon test.

### 3.7. Effect of Donepezil and Rivastigmine on the Concentration of the Reduced Form of GSH Glutathione in Macrophages

#### 3.7.1. Rivastigmine and Donepezil Had No Effect on GSH Concentration in Macrophages

**Figure 6:**
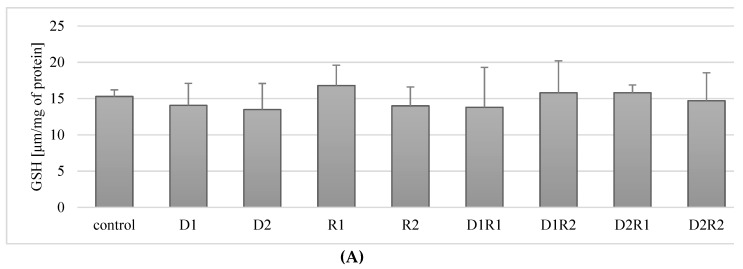

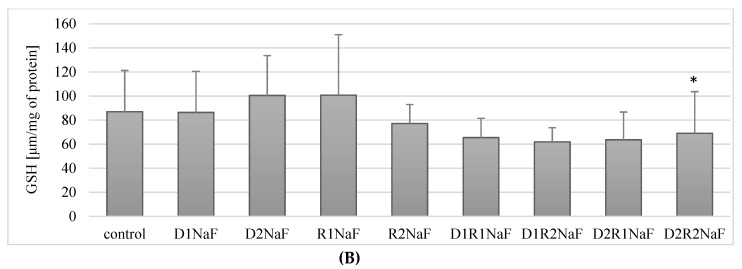
Influence of acetylcholinesterase inhibitors donepezil and rivastigmine on reduced glutathione concentration (GSH) in *THP-1* macrophages (A) and fluoride-exposed *THP-1* macrophages (B).

The exposure of *THP-1* macrophages to donepezil at 20 ng/mL (D1) and 100 ng/mL (D2) did not have a statistically significant effect on GSH concentration compared to controls (*p* = 0.68, *p* = 0.22). No statistically significant changes in GSH concentration were also observed in macrophages exposed to rivastigmine at 5 ng/mL (R1) or 25 ng/mL (R2) compared to controls (*p* = 0.34, *p* = 0.34) (Figure 6A).

In the macrophages cultured with a combination of donepezil and rivastigmine at the lower concentrations (D1R1) did not have a statistically significant effect (*p* = 0.5) on the concentration of GSH in cells, as well as the use of other combinations: D1R2 (*p* = 0.5), D2R1 (*p* = 0.22), D2R2 (*p* = 0.68) (Figure 6A). 

In summary, incubation of macrophages with acetylcholinesterase inhibitors used in separation and in combination (D1R1, D1R2, D2R1, D2R2) did not have a statistically significant effect on the concentration of GSH in cells.

#### 3.7.2. The Combination of Rivastigmine and Donepezil at the Highest Concentrations Reduced the Concentration of the Reduced Form of Glutathione (GSH) in Fluoride-Exposed Macrophages

Incubation of fluoride-exposed macrophages with donepezil at 20 ng/mL (D1) and 100 ng/mL (D2) did not significantly affect the concentration of reduced GSH form compared to the control (*p* = 0.9, *p* = 0.2) (Figure 6B). Exposure to rivastigmine at 5 ng/mL (R1) and 25 ng/mL (R2) also had no effect on GSH concentration (*p* = 0.07, *p* = 0.7) (Figure 6B).

The exposure of macrophages to fluoride and the combinations of donepezil and rivastigmine at 20 ng/mL and 5 ng/mL (D1R1; *p* = 0.2), 20 ng/mL and 25 ng/mL (D1R2; *p* = 0.14) and 100 ng/mL and 5 ng/mL (D2R1; *p* = 0.07) also did not significantly change the concentration of GSH (Figure 5B). However, at the highest concentrations of donepezil at 100 ng/mL and rivastigmine at 25 ng/mL (D2R2) did result in a significant reduction (*p* = 0.04) in GSH concentration by about 20% compared to controls (Figure 6B).

In summary, in fluoride-exposed macrophages, only the combined use of the drugs at the highest concentrations showed a reduction GSH concentration. In the other combinations, there were no statistically significant changes in GSH concentration compared to control.

Donepezil was used at 20 ng/mL (D1) and 100 ng/mL (D2). Rivastigmine was used at 5 ng/mL (R1) and 25 ng/mL (R2). Macrophages incubated with DMSO were used as the control. In a model of fluoride-induced oxidative stress NaF was used at 3 μM and macrophages incubated with NaF and DMSO were used as a control. Cells were cultured with acetylcholinesterase inhibitors for 48 h. After incubation cells were harvested by scraping. GSH concentration was estimated spectrophotometrically using a Glutathione Assay Kit (Cayman Chemical, USA). Data represent means ± SD for 6 independent experiments. * *p* < 0.05, statistically significant differences versus control using Wilcoxon test.

## 4. Discussion

Alzheimer's disease (AD) is one of the most common causes of dementia, and acetylcholinesterase inhibitors (AChE) are the most commonly used drugs to treat this disease. However, no attempt has been made to explain the effect of two popular AChE inhibitors, donepezil and rivastigmine, on the activity of antioxidant enzymes in a model using an agent with a proven pro-oxidant effect. There are only a few studies on the impact of these drugs on the activity of some antioxidant enzymes [60]. This paper is the first attempt to investigate the effect of donepezil and rivastigmine used in concentrations corresponding to the initial and maximum dose of drugs in actual treatment of AD on the activity of antioxidant enzymes and the concentration of glutathione in a model involving the pro-oxidant and inflammation stimulating properties of fluoride in macrophages.

In our experiment, AChE inhibitors used as standard in AD therapy showed possible antioxidant activity in macrophages, inhibiting the formation of ROS, as shown by photos from a confocal microscope. However, under the strong fluoride-induced oxidative stress, the action of the drugs was insufficient, as shown by an increased formation of ROS in the cytoplasm of macrophages, also visible under confocal microscopy.

The observed changes may be attributed to the effect of the studied drugs on the activity of antioxidant enzymes and the concentration of glutathione, which we attempt to explain below.

### 4.1. Acetylcholinesterase Inhibitors-Induced Changes in SOD Activity

The results of studies on the activity of SOD in people with AD are ambiguous. There are reports showing a decrease in SOD activity within the frontal cortex and a slight increase in activity in the caudate nucleus in AD patients, as well as a lack of changes in the activity of this enzyme in AD patients [61,62,63]. Increased activity of the mitochondrial SOD isoform (SOD2) has been reported in the hippocampus of people diagnosed with AD [64,65]. An increase in SOD2 activity has been observed in the area that is usually the most degenerated, i.e. CA1 within the hippocampus [64,65]. The authors suggest that the initially increased activity of antioxidant enzymes in some brain regions in people with AD may be an attempt to compensate for oxidative stress [64,65]. 

Studies on animals show a significant role of SOD in the pathogenesis of AD. Murakami et al., in their studies on mice, demonstrated the potential role of SOD1 downregulation in AD. The researchers drew this conclusion based on cognitive impairment, neuronal inflammation, synaptic protein loss and Tau phosphorylation at Ser-396, oxidative damage or the modulation of soluble Aβ-state [66]. The tests were carried out on mice which were administered, among others, donepezil at a dose of 3mg/kg/month, once a day for 10 days. Administration of donepezil to the mice resulted in an increase in SOD activity in the hippocampus compared to the mice treated with scopolamine alone [66]. 

A slightly different observation was made by Li et al. using also another animal model of AD (intravenous administration of amyloid beta Aβ1-42 to mice). The animals received donepezil intravenously at a dose of 0.01 mg/kg per day but with no effect on the activity of SOD either within the hippocampus or the cerebral cortex [67]. A study conducted on a murine model of AD (intracerebroventricular injection—i.c.v.), showed the ability of rivastigmine to reduce the process of lipid peroxidation in the brain [68].

In our study, the use of AChE inhibitors donepezil and rivastigmine did not significantly affect the activity of SOD. No changes in enzyme activity were noted in cells treated with either a single-drug treatment or in a combination of the drugs at any concentration used. 

AChE inhibitors used in this work are the most preferably chosen drugs with proven efficacy in the treatment of AD. Although the primary reason for the use of AChE inhibitors is the effect on acetylcholine levels, our research and the results of other authors indicate a much wider spectrum of these drugs [16,60]. Activities that may have a beneficial effect on the prevention of disease progression include their effects on antioxidant enzymes [60]. However, our study on THP-1 macrophages has a pioneer character, which makes it difficult to interpret and discuss in the light of other reports. Thus far, no such studies have been conducted on the effects of both drugs. 

We observed a reduced activity of SOD in macrophages under fluoride-induced oxidative stress, treated with donepezil at the lower of the tested concentrations (D1, 20 ng/mL). The reduction of this enzyme's activity was also observed after the addition of rivastigmine at a higher concentration (R2, 25 ng/mL). Current literature indicates the pro-oxidant and suppressive activity of fluoride against antioxidant enzymes (including SOD). Vani et Reddy demonstrated a reduced activity of SOD in the brain and muscle of albino mice receiving NaF (20 mg/kg body weight/day) [69]. The negative effect of fluoride on SOD activity has been described, among others, by Patel and Chinoy, in the murine ovary exposed to fluoride [70]. A similar effect—impairment of SOD activity in the liver, kidneys and heart of mice receiving fluoride—was observed by Sun et al. [71]. A reduction in SOD activity was also described in primary cultured hippocampal neurons cultured with NaF [72]. However, the effect of AChE inhibitors on SOD in fluoride-exposed macrophages has never been investigated, and the results presented in our work are completely novel.

### 4.2. Catalase (CAT) Activity Alterations in Response to Inhibitors of Acetylcholinesterase

The results obtained in our study show that the separately used anti-Alzheimer drugs increased the activity of catalase (CAT) in the macrophages. An increase in enzyme activity was observed for each concentration of the drugs used, i.e. for donepezil used at 20 ng/mL (D1) and 100 ng/mL (D2) and for rivastigmine at 5 ng/mL (R1) and 25 ng/mL (R2). However, in the cases where the drugs were used together, no statistically significant variations in CAT activity were observed. 

In current literature, in a group of AD patients, Klugman et al. showed that the use of AChE inhibitors did not significantly affect the activity of catalase in patients taking medications compared to the so-called drug-naive patients [73]. However, research conducted by Zhang and Tang on rat pheochromocytoma line PC12 shows that pretreatment of cells with donepezil (10 μM) before exposure to H_2_O_2_ led to improved cell survival, and enhanced antioxidant enzymes activities (including catalase). According to the authors, the neuroprotective effect of the drugs resulting from their antioxidative activity could partly be responsible for the clinically observed efficacy of these preparations [74]. The results of studies on the effect of AChE on CAT activity are ambiguous. In a mouse AD model (induction of disease caused by scopolamine), administration of donepezil 5 mg/kg once a day for nine days prior to scopolamine administration resulted in increased CAT activity within whole brain lysate [75]. The authors concluded that the drugs they used, including donepezil, resulting in the increased activity of antioxidant enzymes (including CAT), weakened peroxidation and showed anti amnesic activity due to the decreased activity of AChE [75].

In our model of fluoride-induced oxidative stress, a decrease in CAT activity was observed in macrophages incubated with a combination of donepezil and rivastigmine at 100 ng/mL and 5 ng/mL, respectively (D2R1). In the other experimental conditions, no significant effect of AChE inhibitors on CAT activity was observed.

So far, little research has been done on the effects of AChE inhibitors on CAT activity, and the results obtained have varied. This paper is the first in which an attempt was made to study the influence of AChE inhibitors on fluoride-exposed macrophages at given concentrations. In recent years, attention has been increasingly focused on the role of fluoride in the pathogenesis of oxidative stress, and the mechanism of this phenomenon is explained both by the effect of this element on ROS and the direct activity of antioxidant enzymes themselves, including CAT [76]. The most frequently described effect caused by fluoride is the inhibition of catalase activity, as demonstrated in studies on human and animal tissues [50,77,78]. The exact mechanism of the action of fluoride on enzyme activity is still being investigated. However, it is being currently suggested that the inhibitory effect of fluoride on CAT activity results from the F^−^ ability to interact with the metal ions (including tri- as well divalent ions) situated within the antioxidant enzymes catalytic site. Described interaction may possibly result in the enzymes (counting CAT) inhibition [79,80].

### 4.3. The Effect of Acetylcholinesterase Inhibitors on the Activity of Glutathione Peroxidase (GPx), Glutathione Reductase (GR) and the Concentration of the Reduced Form of Glutathione (GSH)

In our study, macrophage exposure to donepezil and rivastigmine did not cause changes in GPx activity. No effect on enzyme activity was observed for either the drugs used separately (at lower and higher concentrations: D1, D2, R1, R2) or for the drugs used in combination (D1R1, D1R2, D2R1, D2R2). After the use of donepezil and rivastigmine (separately and in all combinations), there were no changes in the activity of glutathione reductase. In addition, no changes in the concentration of the reduced form of glutathione (GSH) were observed after the use of the drugs. 

As has already been mentioned, for some time researchers have emphasized the need for a thorough investigation of drugs used in Alzheimer's disease (AD) on the parameters of the body's antioxidant system [60]. However, no study results have been published describing the effect of donepezil and rivastigmine on GR and GPX activity and glutathione levels. In the current reports, only a few items can be found regarding the influence of AChE inhibitors on the antioxidative system associated with glutathione [60]. 

The value of the current work seems to be all the more important due to the fact that current studies on the influence of AChE inhibitors on GPx activity were carried out primarily in an animal model of AD. Klugman et al. demonstrated that AD patients receiving AChE inhibitors did not show any change in GR activity compared to the drug-naive group of patients with AD [73]. Gubandru et al. showed that in patients with AD, receiving rivastigmine or donepezil with memantine did not significantly affect the concentration of GSH [81]. Li et al. observed that in mice with Aβ–induced AD, the administration of donepezil 0.01 mg/kg/day (ICV) resulted in an increased concentration of GSH in the hippocampus and cerebral cortex, and the increased activity of GPx [67]. An increase in GSH within the hippocampus was also confirmed by Hou et al. In a model of AD using transgenic animals, a decrease in GSH concentration was observed after a 16-week treatment with donepezil at a dose of 2.5 mg/kg [82]. A decrease of GSH concentration was also described by Kumar et al. after administration of galantamine, and the decrease was accompanied by increased GPx activity [83]. Khurana et al., who conducted studies on a rat model of AD, administering rivastigmine at 2.5 mg/kg for 28 days, followed by colchicine, did not cause any changes in GSH concentration in the brains of the rats studied [84]. 

In our study we found no changes in GPx activity in fluoride-exposed macrophages compared to controls. However, a decrease in GR activity was observed in cells incubated with donepezil at 20 ng/mL with rivastigmine at 25 ng/mL (D1R2), and donepezil at 100 ng/mL with rivastigmine at 5 ng/mL (D2R1), and donepezil at 100 ng/mL with rivastigmine at 25 ng/mL (D2R2). 

In our model of fluoride toxicity, we found only a decrease in the concentration of GSH in macrophages incubated with donepezil at 100 ng/mL and rivastigmine at 25 ng/mL (D2R2). As mentioned above, this paper is the first to attempt to investigate the influence of the most commonly used AChE inhibitors on the activity of antioxidant enzymes in the model of fluoride toxicity on macrophages. Although, as in the case of the previously described components of the enzyme antioxidant system, the effect of fluoride on the activity and concentration in the case of GPx, GSH and GR varies, the most often mentioned is the fluoride-induced inhibition of the activity of these enzymes, a reduction in GSH and SOD levels, as well as increased lipid peroxidation in rats receiving sodium fluoride in drinking water [85]. The pro-oxidant effect of fluoride, its inhibitory effect on GPx activity, and a reducing effect on GSH concentration have also been described by Inkielewicz et al. [86].

### 4.4. Potential Mechanism of Inhibitory Effects of Fluoride on Acetylcholinesterase Inhibitors

The results of this study show that the AChE inhibitors donepezil and rivastigmine had a different effect on the activity of antioxidant enzymes and GSH concentration in macrophages not exposed to fluoride compared to our model of fluoride-induced oxidative stress. This observation is in agreement with the results of our previous study on the influence of AChE inhibitors on the activity and expression of cyclooxygenases in the same model of fluoride toxicity.

In the present study, AChE inhibitors increased CAT activity or did not affect the activity of SOD, GPx, GR and GSH concentration in macrophages not exposed to fluoride. In contrast exposure to fluoride and AChE inhibitors resulted in a decrease in CAT, SOD, GR and GSH concentrations. Therefore, exposure to a pro-oxidant agent (such as fluoride used in our model) seems to be a factor that can modulate or even cancel the antioxidant effect of AChE inhibitors.

AChE inhibitors have been repeatedly shown to decrease neurotoxicity associated with the action of β-amyloid peptide in AD. Interestingly, one of the suggested mechanisms of β-amyloid peptide neurotoxicity is pro-oxidant activity, just as in the case of fluoride [87]. Inhibition of β-amyloid peptide is most likely associated with the mechanism of upregulation and higher expression of α subtypes (7 along with 3) [88] with the stimulation of α7 l nicotinic acetylcholine receptors of neurons (nAChRs) belonging to ligand-gated ion channels, key for learning and memory [89,90,91], and defending against the β-amyloid peptide toxicity [92]. On the other hand, fluorosis does not result in a change of α7 subunit at the mRNA level in the rat brain [93].

In this study, the antioxidant effect of AChE inhibitors seemed to be suppressed in the presence of fluoride, resulting in the inhibition of antioxidant enzyme activity and reduced GSH concentration. This is in line with the results obtained by Goschorska et al. and Gutowska et al. in their studies on the same model, showing that the fluoride-induced overproduction of ROS [28,94] resulted in phosphorylation [95] and elevated activity JNK1/2, MAPK ERK1/2 or p38 [96,97,98], presumably via the tyrosine kinase stimulation together with simultaneous tyrosine phosphatases suppression [99]. These results are all the more significant as MAP kinases are mentioned in literature as enzymes with a particularly high redox sensitivity [100].

Although fluoride does not affect expression of the 7α subunit at the position of mRNA in the rats’ cerebrum, it does affect the signaling pathways associated with the activation and activity of MAP kinases. Activation of MAPK is one of the first mechanisms of fluoride's neurotoxic action (including pro-oxidant) in the CNS [77], with particular severity observed in the hippocampus [101]. In response to the mentioned properties exhibited by fluoride, the RAS-stimulated reactions are accelerated. As the consequence of the above discussed interactions, the activation of MEK/MEKK and further ERK (extracellular signal-regulated protein kinase) enhancement occurs [102]. Activation of Ras, resulting from increased peroxidation, induces recruitment of phosphatidylinositol 3'- kinase to Ras, which is essential for the further activation of Akt and MAP [103].

It is possible that a MAPK-dependent pathway is responsible for the inhibitory effect of fluoride on the pro-oxidant action of AChE inhibitors. Stimulating the 7α nAChR subunit, donepezil and rivastigmine affect the activation of mainly two pathways: the phosphatidylinositol 3-kinase-Akt signaling pathway and the MAPK pathway [104,105]. The effect on the MAPK-dependent pathway is associated with the positive effect of donepezil on the parameters of Clinical Dementia Rating used to assess the status of patients with dementia [106].

This paper, based on proven pro-oxidative and pro-inflammatory effects of fluoride, is the first attempt to demonstrate the influence of environmental factors (such as fluoride) on the action of two most frequently and widely used acetylcholinesterase inhibitors at concentrations corresponding to the actual doses of drugs (the lowest and maximum therapeutic doses). The results of this study are extremely interesting and important from the scientific and clinical perspective. On the one hand, they suggest that these drugs, used so widely in developed countries, may stimulate the activity of catalase (CAT) or have no significant effect on the activity of antioxidant enzymes or GSH. On the other hand, under conditions of severe stress resulting from the presence of fluoride, they lowered the activity of some antioxidant enzymes.

## 5. Conclusions

Donepezil and rivastigmine are considered to be the most effective and most commonly used AChE inhibitors during Alzheimer's disease pharmacotherapy. For a long time, attention has also been paid to their immunomodulatory, anti-inflammatory and antioxidant properties. The latter effect, including the effect on antioxidant enzymes and GSH concentration, has been the least researched. In addition, studies in this field have been carried out in different models (mainly rodents), and usually concerned one of the drugs and only selected antioxidant enzymes.

This work is the first attempt to demonstrate the effect of fluoride-induced oxidative stress on the antioxidant action of two most common and widely used AChE inhibitors, donepezil and rivastigmine, at concentrations corresponding to the actual doses of the drugs used in clinical practice. On one hand, we found that these drugs stimulated catalase (CAT) activity in THP-1 macrophages but had no significant effect on the activity of other antioxidant enzymes or GSH concentration. However, when the macrophages were exposed to fluoride and rivastigmine and/or donepezil, we observed a decrease in the activity of CAT, SOD and GR. This observation suggests that the fluoride-induced oxidative stress may suppress the antioxidant action of AChE inhibitors.

As mentioned above, methanesulfonyl fluoride, which irreversibly and selectively inhibits brain acetylcholinesterase, has been taken under consideration in AD treatment. Therapeutic positive effects seem to exceed those exerted by the most commonly used and approved medications [36]. In relation to the information above, it is not excluded that low (3µM) concentration of NaF would also exert the inhibition of acetylcholinesterase. Thus, further research concerning this aspect seems to have significant importance. 

Our results may have significance in the clinical practice of treatment of AD and other dementia diseases, by neurologists, psychiatrists, geriatricians, internists and general practitioners, as they suggest that oxidative stress may suppresses any potential antioxidant effect of AChE inhibitors. They also point to the need for further research in this direction.

## Figures and Tables

**Table 1:** Concentrations of the drugs used in the experiment. The concentrations were based on the concentrations within the serum of the persons that receive the minimal and maximum therapeutic doses. Drugs were dissolved in dimethyl sulfoxide (DMSO).

Symbol	Drug	Concentration (ng/mL)
**D1**	donepezil	20
**D2**	donepezil	100
**R1**	rivastigmine	5
**R2**	rivastigmine	25

**Table 2:** Diagram of experiment of macrophages exposed to donepezil and rivastigmine.

Group	Procedure	Donepezil Concentration	Rivastigmine Concentration
**Control**	macrophages cultured with DMSO	-	-
**D1**	macrophages cultured with donepezil	20 ng/mL	-
**D2**	macrophages cultured with donepezil	100 ng/mL	-
**R1**	macrophages cultured with rivastigmine	-	5 ng/mL
**R2**	macrophages cultured with rivastigmine	-	25 ng/mL
**D1R1**	macrophages cultured with both medicaments	20 ng/mL	5 ng/mL
**D1R2**	macrophages cultured with both medicaments	20 ng/mL	25 ng/mL
**D2R1**	macrophages cultured with both medicaments	100 ng/mL	5 ng/mL
**D2R2**	macrophages cultured with both medicaments	100 ng/mL	25 ng/mL

**Table 3:** Schematic presentation of experiments on macrophages treated with sodium fluoride and the drugs donepezil and rivastigmine dissolved in DMSO.

Group	Procedure	NaF Concentration	Donepezil Concentration	Rivastigmine Concentration
**Control**	macrophages cultured with NaF and DMSO	3 µM	-	-
**D1NaF**	macrophages cultured with donepezil and NaF	3 µM	20 ng/mL	-
**D2NaF**	macrophages cultured with donepezil and NaF	3 µM	100 ng/mL	-
**R1NaF**	macrophages cultured with rivastigmine and NaF	3 µM	-	5 ng/mL
**R2NaF**	macrophages cultured with rivastigmine and NaF	3 µM	-	25 ng/mL
**D1R1NaF**	macrophages cultured with donepezil, rivastigmine and NaF	3 µM	20 ng/mL	5 ng/mL
**D1R2NaF**	macrophages cultured with donepezil, rivastigmine and NaF	3 µM	20 ng/mL	25 ng/mL
**D2R1NaF**	macrophages cultured with donepezil, rivastigmine and NaF	3 µM	100 ng/mL	5 ng/mL
**D2R2NaF**	macrophages cultured with donepezil, rivastigmine and NaF	3 µM	100 ng/mL	25 ng/mL

**Table 4:** Donepezil (D) and/or rivastigmine (R) influence on intracellular ROS synthesis in macrophages obtained from the *THP-1* monocytic cell line or in macrophages exposed to rivastigmine and/or donepezil in a model of fluoride-induced oxidation.

Experimental Conditions	(D) and/or (R)		(D) and/or (R) + NaF	
	DCF Fluorescence Intensity ^#^	% Decrease/Increase vs. Control	DCF Fluorescence Intensity ^#^	% Decrease/Increase vs. Control
C (*n* = 6)	39.87 ± 1.51		46.58 ± 1.78	
D1 (*n* = 6)	38.25 ± 1.17	−4.06	52.26 ± 1.96	12.19 *
D2 (*n* = 6)	38.76 ± 2.76	−2.78	52.45 ± 2.02	12.60 *
R1(*n* = 6)	39.95 ± 3.43	−0.20	54.67 ± 3.67	17.36 *
R2 (*n* = 6)	41.13 ± 1.15	3.16	55.32 ± 1.43	18.76 *
D1R1(*n* = 6)	40.01 ± 1.24	0.35	51.43 ± 2.11	10.41 *
D1R2 (*n* = 6)	40.02 ± 1.14	0.38	50.55 ± 1.32	9.93 *
D2R1(*n* = 6)	39.55 ± 1.22	−0.80	52.21 ± 2.67	12.08 *
D2R2 (*n* = 6)	39.85 ± 2.54	−0.05	50.02 ± 1.54	7.39 *

* *p* < 0.005, significant difference vs control (Mann–Whitney test). ^#^ normalized to total protein levels
